# Effect of Pre-Exposure to Deoxynivalenol on the Response of Porcine Intestinal Epithelial Cells to F18 *E. coli* Infection

**DOI:** 10.3390/toxins18030141

**Published:** 2026-03-14

**Authors:** Madison Brackett, Paul Oladele, Hang Lu, Nathan Horn, Kolapo M. Ajuwon

**Affiliations:** 1 Department of Animal Sciences, Purdue University, West Lafayette, IN 47907, USA; land5@purdue.edu (M.B.); poladele@purdue.edu (P.O.); 2 United Animal Health, Sheridan, IN 46069, USA; hang.lu@unitedanh.com (H.L.); nathan.horn@unitedanh.com (N.H.)

**Keywords:** gastrointestinal barrier, deoxynivalenol, enterotoxigenic *E. coli*, IPEC-J2, pig, adhesion

## Abstract

The mycotoxin deoxynivalenol (DON) is a common contaminant found in swine diets, causing decreased growth performance and poor health. Additionally, F18 enterotoxigenic *E. coli* is a leading cause of post-weaning diarrhea. Nursery pigs are often exposed to each of them after weaning; however, it is unknown what impact the combination of these stressors has on gastrointestinal health. Therefore, the objective of this study was to investigate the effect of pre-exposure to DON on the response of intestinal porcine epithelial cells (IPEC-J2) to challenge with enterotoxigenic F18 *E. coli*. Four groups were compared: Control (untreated cells), DON (cells treated with 0.5 μM DON for 24 h), F18 *E. coli* (multiplicity of infection 5:1, varied duration) and DON + *E. coli* (DON treatment with subsequent *E. coli* infection). Gene expression of IL-8, IL-6 and TNFα was significantly increased in cells infected with *E. coli* for 3 h vs. uninfected cells (*p* < 0.0001, *p* < 0.0001 and *p* < 0.0001, respectively). There was an interactive effect between DON and *E. coli* on IL-8 gene expression; cells pretreated with DON before *E. coli* infection had a higher expression of IL-8 than those not pretreated (*p* < 0.05). The concentration of IL-8 protein was significantly increased by *E. coli* (*p* < 0.0001). Claudin 1 and Occludin protein abundance were reduced by *E. coli* as measured by Western blot. Cytotoxicity was increased by *E. coli* vs. Control (*p* < 0.05). Pretreatment with DON increased the amount of *E. coli* that adhered to IPEC-J2 cells (*p* < 0.01) 30 min post-infection. FITC-dextran passage was increased in the DON + *E. coli* treatment vs. *E. coli* alone (*p* < 0.0001). Transepithelial electrical resistance (TEER) was decreased by DON when compared to untreated cells at 0 h (*p* < 0.0001). Similarly, DON + *E. coli* exhibited lower TEER vs. *E. coli* alone at 2 h post-infection (*p* < 0.0001). Taken together, these results indicate that DON pre-exposure increased the severity of *E. coli* infection on endpoints such as barrier permeability and *E. coli* adhesion.

## 1. Introduction

The gastrointestinal tract is responsible for nutrient digestion and absorption, as well as maintaining a barrier between luminal contents and internal bodily systems [1]. The gastrointestinal epithelial barrier is the first line of defense to protect the animal from potentially harmful feed contaminants [2]. This barrier is protected by tight-junction proteins, mucins, and robust immune surveillance [3]. Disruption of the barrier between intestinal cells can lead to decreased nutrient absorption, gut leakiness, and increased pathogen or toxin invasion [4,5]. In addition to barrier maintenance, immune surveillance of the gastrointestinal tract is important to protect the host from pathogens and toxins. If activated, a key part of the innate immune response is the secretion of inflammatory mediators. Mediators such as pro-inflammatory cytokines signal for further immune cell recruitment as needed. However, if left unchecked, inflammation can have negative effects on the animal, including the impaired function of the gastrointestinal barrier or damaged tissues.

Deoxynivalenol (DON) is a trichothecene mycotoxin that is primarily produced by fungi belonging to the *Fusarium* species. DON is regularly found contaminating swine diets and is known for its negative effects on growth performance and health. Of the species that may be exposed to DON, pigs are particularly sensitive [6]. Relatively low levels of DON in the diet have proven to negatively impact growth performance and gut morphology [7]. Additional evidence suggests that DON harms the gut of pigs by increasing the permeability of the gastrointestinal lining, decreasing mucus production, increasing oxidative stress, and altering gut morphology [6]. Previous research shows that DON exposure can decrease cell viability and cause apoptosis [8,9,10]. Various in vitro studies suggest that DON time- and dose-dependently reduces the strength of the epithelial barrier as observed by increases in the permeability of FITC-dextran and decreases in transepithelial electrical resistance [11,12,13]. Other cell-based studies report that DON exposure decreases tight-junction protein expression [14,15]. In addition to disrupting the intestinal barrier, DON has been found to induce inflammation in vitro [16,17]. However, other investigations of DON did not observe an increase in inflammation. This indicates that DON likely induces inflammation in a context-dependent manner, affected by its dose and duration of exposure [6].

*Escherichia coli* (*E. coli*) is well-established as a leading cause of post-weaning diarrhea in piglets [18,19]. Furthermore, enterotoxigenic *E. coli* (ETEC) infections can lead to depressed growth performance parameters [20]. Distinguished by their fimbriae, F18 and F4 are the most common types of ETEC found commercially. The attachment of *E. coli* allows for colonization and subsequent release of heat-labile and heat-stable toxins, which disrupt the electrolyte channels of cells. ETEC has been shown to decrease tight-junction gene and protein expression of cells [21,22,23,24], increase FITC-dextran passage [25,26,27], decrease TEER [28,29,30], and increase expression of pro-inflammatory cytokines [22,31,32].

Due to the high prevalence of DON and ETEC in the commercial swine industry, it is likely that many young pigs experience both challenges concurrently. While we have provided evidence that supports the independent effects of DON and ETEC on the intestinal epithelial barrier, there is very limited research on the effects of DON and ETEC in combination. DON-induced barrier damage, oxidative stress, and inhibition of protein synthesis were suspected to leave cells more vulnerable to ETEC infection. We hypothesized that pre-exposure to DON would increase the severity of *E. coli* infection by influencing the strength of the epithelial barrier and inflammatory markers. Therefore, the objective of this study was to investigate the effect of DON pre-exposure on IPEC-J2 cell response to a subsequent *E. coli* infection.

## 2. Results

### 2.1. E. coli Infection Regulated Changes in Expression of Genes Involved in Inflammatory, Antioxidant and Metabolic Response Pathways

**Figure 1:**
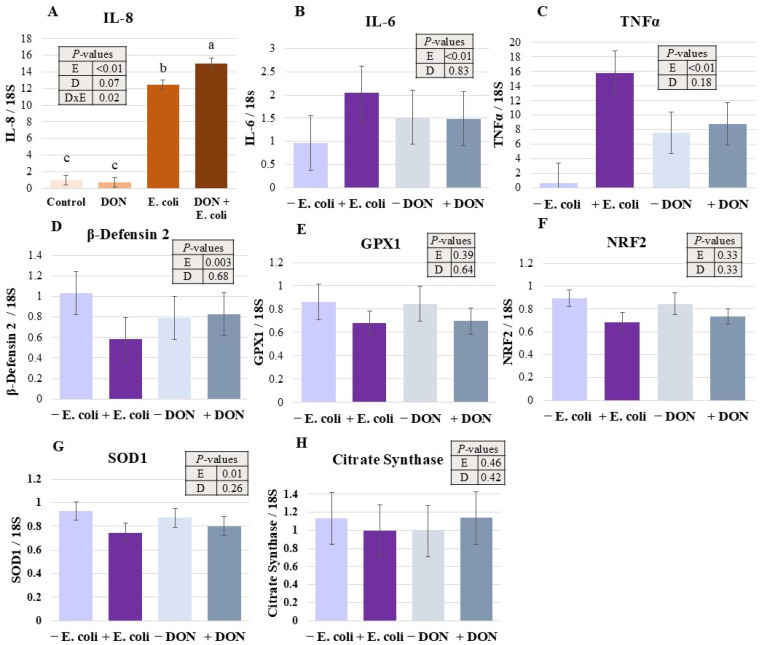
Effects of DON and *E. coli* on the relative gene expression of inflammatory, antioxidant, and metabolic parameters in IPEC-J2 cells. Pro-inflammatory markers included IL-8 (**A**), IL-6 (**B**), and TNFα (**C**). β-Defensin 2 (**D**) was measured as an anti-inflammatory marker. Antioxidant markers included GPX1 (**E**), NRF2 (**F**), and SOD1 (**G**). Citrate synthase (**H**) was utilized as a metabolic parameter. Data on GPX1 and NRF2 were abnormal and were tested using the Kruskal–Wallis test. Each panel’s number of observations (*n*) are as follows: (**A**), 5–6; (**B**), 12; (**C**), 8–11; (**D**), 12; (**E**), 12; (**F**), 12; (**G**), 12; (**H**) 9–11. Letters denote significant pairwise differences (*p* ≤ 0.05). *p*-values are representative of PROC-MIXED results, and *E. coli* (E) and DON (D) main effects are represented. Significant interaction of DON and *E. coli* (D × E) is included as applicable. Superscript letters a, b and c indicate significant mean differences (*p* < 0.05).

The relative expression of genes involved in inflammatory, antioxidant, and metabolic response pathways is presented in Figure 1. The pro-inflammatory chemokine IL-8 increased more than 10-fold as a result of *E. coli* treatment (*p* < 0.0001, Figure 1A). Furthermore, DON and *E. coli* interacted to increase IL-8 gene expression (*p* < 0.05). Through pairwise comparisons, it was found that the gene expression of IL-8 in the combination of DON + *E. coli* treatment was significantly increased relative to *E. coli*, DON, and Control treatments (*p* < 0.05). Additionally, the *E. coli* treatment was higher than DON and Control treatments (*p* < 0.05, Figure 1A). The expression of IL-6 and TNFα genes was increased due to *E. coli* treatment (*p* < 0.0001 and *p* < 0.0001, respectively; Figure 1B,C). *E. coli* infection downregulated the gene expression of β-Defensin 2 (*p* < 0.01, Figure 1D). The expression of SOD1 was also reduced by *E. coli* (*p* = 0.01, Figure 1D). The gene expression of GPX1, NRF2, and citrate synthase was unaffected by treatments (Figure 1E, Figure 1F and Figure 1H, respectively).

### 2.2. E. coli Challenge Increased Abundance of IL-8 Protein

**Figure 2:**
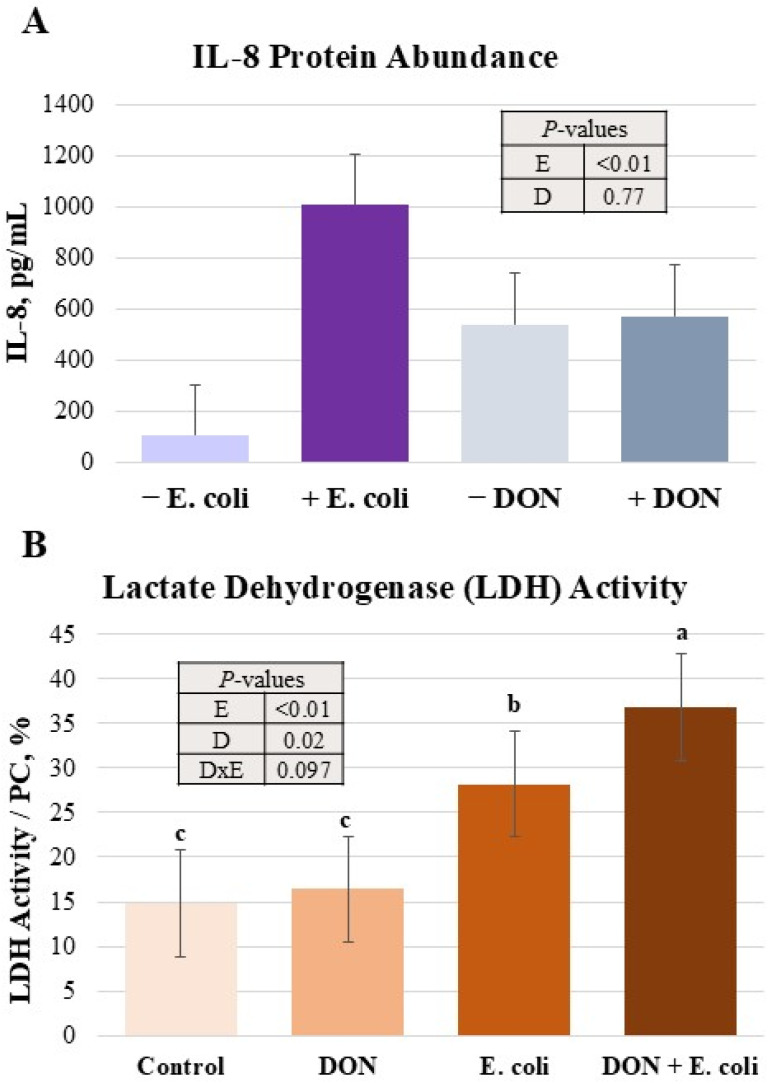
Effects of DON and *E. coli* on IL-8 protein abundance and lactate dehydrogenase activity. IL-8 protein abundance (**A**) was measured as a pro-inflammatory marker. Lactate dehydrogenase activity (**B**) indicated the effects of treatments on relative cytotoxicity. Each panel’s number of observations (*n*) are as follows: (**A**), 10; (**B**), 5–6. *p*-values are representative of PROC-MIXED results, and *E. coli* (E) and DON (D) main effects are represented. Significant interaction of DON and *E. coli* (D × E) is included as applicable. Superscript letters a, b and c indicate significant mean differences (*p* < 0.05).

Due to the observed increases in IL-8 gene expression in some treatments, IL-8 protein concentration was measured (Figure 2A). There was a 10-fold increase in IL-8 protein abundance from the *E. coli* challenge (*p* < 0.0001). There was no impact of DON, nor an interaction between DON and *E. coli*.

### 2.3. E. coli and DON Increased IPEC-J2 Cytotoxicity

Measurement of LDH activity as an indicator of cell death is presented in Figure 2B. *E. coli* infection increased (*p* < 0.0001) LDH activity. The combination of DON and *E. coli* tended (*p* = 0.06) to but did not significantly interact to increase LDH activity. Pairwise comparisons showed LDH activity was elevated in DON + *E. coli* compared to *E. coli*, DON, and Control treatments (*p* < 0.05) alone. The *E. coli*-alone treatment was also higher in LDH activity than DON and Control treatments (*p* < 0.05).

### 2.4. Tight-Junction Protein Expression Was Differentially Impacted by Treatments

**Figure 3:**
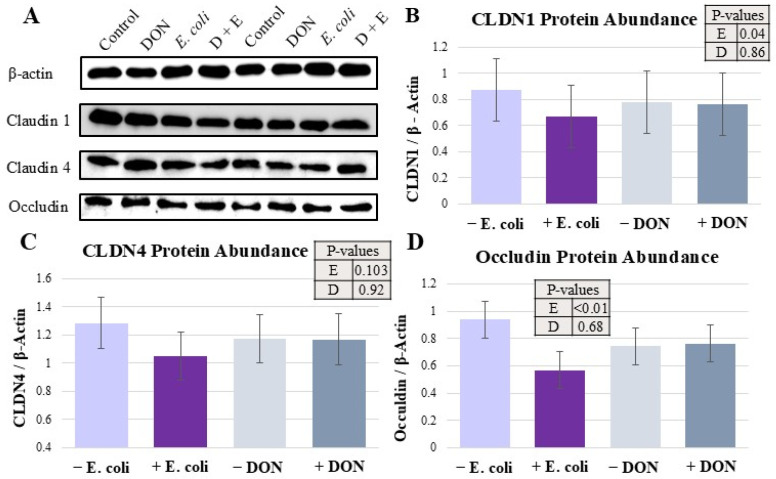
Effects of DON and *E. coli* on tight-junction protein abundance as obtained through Western blot (**A**). Claudin 1 (**B**), Claudin 4 (**C**), and Occludin (**D**) protein expressions were quantified relative to β-actin. Each panel’s number of observations (*n)* are as follows: (**B**), 13; (**C**), 16; (**D**), 16. *p*-values are representative of PROC-MIXED results, and *E. coli* (E) and DON (D) main effects are represented.

The abundance of tight-junction proteins as determined by Western blots is shown in Figure 3. Claudin 1 abundance was downregulated by *E. coli* treatment (*p* < 0.05, Figure 3B). Occludin level was also decreased by *E. coli* (*p* < 0.0001, Figure 3D). Claudin 4 protein expression was numerically reduced but not significantly altered by treatments (Figure 3C).

### 2.5. Cells Pre-Exposed to DON Demonstrated an Impaired Epithelial Barrier and Elevated E. coli Attachment

**Figure 4:**
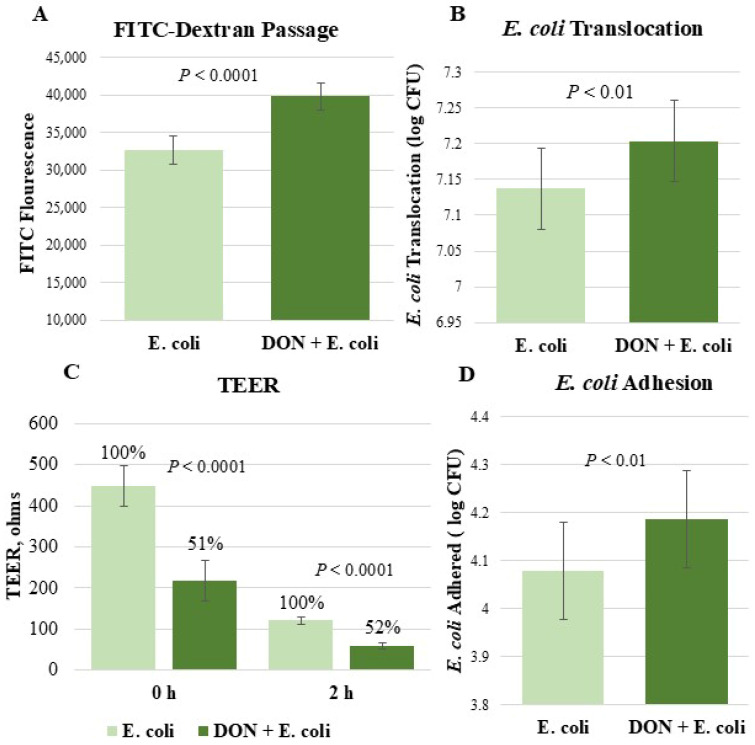
Effect of DON and *E. coli* on epithelial barrier integrity and susceptibility to *E. coli* adhesion. The amount of FITC-dextran (**A**) and *E. coli* (**B**) to pass through the cell monolayer was measured. TEER (**C**) was measured prior to *E. coli* infection but after 24 h of DON exposure (0 h) and two hours post-infection with *E. coli* (2 h). The amount of *E. coli* that adhered to IPEC-J2 cells was also quantified (**D**). Each panel’s number of observations (*n*) are as follows: (**A**), 8–9; (**B**), 6–7; (**C**), 8–9; (**D**), 12.

Because of the known effects of DON and *E. coli* on epithelial barrier integrity, *E. coli* translocation, FITC-dextran passage, and TEER experiments were performed (Figure 4A–C). After 2 h of *E. coli* infection, passage of FITC-dextran into basolateral media was increased by 22% in the treatment pre-exposed to DON (*p* < 0.0001, Figure 4A). Furthermore, *E. coli* translocation was higher in the DON + *E. coli* treatment than *E. coli* alone (*p* < 0.01, Figure 4B). Likewise, prior to *E. coli* infection but after 24 h of DON exposure, there was a significant reduction in TEER (51%) when comparing untreated cells to cells exposed to DON (*p* < 0.0001, Figure 4C). Two hours after *E. coli* infection, DON + *E. coli* treatment had a 48% lower TEER value relative to *E. coli* alone (*p* < 0.0001).

The amount of *E. coli* that attached to IPEC-J2 cells was measured to consider the effect of DON on subsequent *E. coli* attachment (Figure 4D). There was a higher level of *E. coli* adhesion in the DON + *E. coli* treatment relative to *E. coli* alone (*p* < 0.01).

## 3. Discussion

Mycotoxins, particularly DON, are toxins that contribute to widespread feed contamination in the swine industry [33,34]. Additionally, F18 ETEC is routinely detected in cases of post-weaning diarrhea, making it one of the most relevant pathogens facing young pigs [18]. Because DON and ETEC are highly prevalent in the commercial swine industry, it is likely that a significant number of pigs are exposed to both stressors simultaneously. Evidence supports the independent effects of DON and ETEC on the gastrointestinal system of pigs. However, there is no research considering how DON may exacerbate the response of piglets to F18 ETEC infection. Here, we utilized IPEC-J2 cells as a model to determine if the negative effects of DON increase pigs’ vulnerability to the adverse effects of F18 ETEC in the gastrointestinal tract.

Inflammation is a part of the innate immune system that is important for protecting the host from immune threats. In the present study, it was found that the gene expression of the pro-inflammatory cytokines IL-8, IL-6, and TNFα was elevated by the *E. coli* challenge. Upregulation of pro-inflammatory cytokine gene expression following ETEC infection is consistent with previous studies [32,35,36]. This response can be attributed to the activation of Toll-like receptor and NF-kB pathways, which are a known part of the immune response to *E. coli* infection [37]. The gene expression of β-Defensin 2, an antimicrobial peptide, was decreased by *E. coli*. A study found that immediately following ETEC infection, there was an increase in β-Defensin 2 gene expression, followed by a decrease at 2 h and another increase at 4 h post-infection [38]. Gene expression can be highly transient to ensure proper regulation of translation of certain proteins. Therefore, the decrease in β-Defensin 2 observed 3 h post-infection in this study may be due to the cells’ attempt to regulate its protein abundance after a sharp increase in expression immediately following *E. coli* infection. While *E. coli* had a robust effect on inflammatory markers, DON had a limited impact. It was observed that DON interacted with *E. coli* to increase IL-8 gene expression. However, this effect of DON did not carry through to IL-8 protein abundance. Other investigations observed a DON-induced increase in pro-inflammatory markers at higher concentrations of DON than used for this study [9,39,40]. Thus, the contrasting results of DON-induced inflammation are likely due to the discrepancy in doses [8]. Under the current experimental conditions, the changes in gene expression of inflammatory markers did not appear to be exacerbated by DON and were driven primarily by *E. coli* infection.

As intestinal epithelial cells are essential for gastrointestinal barrier function, their health is vital to gut homeostasis. Thus, intestinal cell death or impaired viability can have detrimental consequences on the performance of the gastrointestinal tract [41]. In agreement with previous studies, it was observed that DON and *E. coli* increased cell death as indicated by elevated LDH activity compared to untreated cells [42]. The increase in LDH activity is indicative of cellular stress that may signal apoptosis, necrosis, or cell membrane damage [42,43]. Both DON and *E. coli* have been found to dose-dependently increase apoptosis in cells [44,45,46,47]. Several previous reports corroborate the observed effects of DON on cell viability [8,9]. Mechanistically, DON has the capacity to increase reactive oxygen species (ROS) and decrease antioxidant expression, leading to oxidative stress and ultimately apoptosis [8]. Additionally, DON has been demonstrated to activate genes involved in necroptosis pathways [12,40]. *E. coli* has a well-established negative effect on cell viability [46,47]. One signaling pathway that could be responsible for decreasing cell viability following ETEC infection is inhibition of the PI3K–AKT pathway, which plays roles in protein synthesis, cell growth and cell survival [48,49]. Furthermore, toxins from *E. coli* have been shown to increase ROS and decrease antioxidant activity, damage DNA and, by extension, result in apoptosis [47]. In the current study, we observed a tendency for DON and *E. coli* to interact to increase cell death. While the literature is robust concerning the effects of DON or *E. coli* alone on cell viability, there is a gap in research regarding how they may synergistically increase cell death. It is possible that the combination of DON and *E. coli* triggers severe oxidative stress that overwhelms all antioxidant defenses and leads to an increase in apoptosis. Alternatively, the inhibition of protein synthesis by DON may cause cells to be unable to effectively create mediators to defend against *E. coli*-induced damage, increasing the incidence of cell death. However, more studies would be needed to elucidate the exact mechanism behind the further increase in cell death in the combination DON and *E. coli* treatment. Overall, these findings suggest that cells exposed to DON prior to *E. coli* infection were more susceptible to cell death than cells in the DON or *E. coli* treatments alone.

Proper expression of tight-junction proteins is essential for maintaining epithelial barrier integrity. In this study, the expression levels of Claudin-1, Claudin-4, and Occludin were measured to assess the effects of DON and *E. coli* on tight-junction integrity. Consistent with previous findings, *E. coli* challenge reduced the expression of Claudin-1 and Occludin [50]. This reduction may be associated with activation of the NF-κB signaling pathway following ETEC infection, which is known to suppress tight-junction protein expression [51,52]. Additionally, toxins produced by *E. coli* may contribute to tight-junction disruption, although the exact mechanisms remain unclear [53]. Oxidative stress induced by *E. coli* may also play a role in downregulating tight-junction protein expression [54].

In contrast, DON did not significantly affect the expression of the tight-junction proteins measured in this study. Previous reports have shown reductions in tight-junction protein expression at higher DON concentrations, suggesting that the relatively low dose used in the present study resulted in the lack of any effect [13,15,55]. Overall, these findings indicate that *E. coli* is a primary driver of tight-junction disruption, and DON pre-exposure did not exacerbate this specific effect.

Barrier permeability was evaluated by measuring the passage of 4 kDa FITC-dextran and *E. coli* across the IPEC-J2 cell monolayer. Pre-exposure to DON increased FITC-dextran permeability by 22% and enhanced *E. coli* translocation, indicating aggravated barrier dysfunction when both stressors were combined. These observations are consistent with previous studies demonstrating that both DON and *E. coli* independently compromise barrier integrity [15,25,27,39,56]. The increased permeability observed with DON exposure may be attributed to cell death, tight-junction dysregulation or mislocalization, and oxidative damage [54,57,58].

To further measure the effect of treatments on barrier integrity, TEER was measured. Prior to *E. coli* infection but after 24 h of DON challenge, IPEC-J2 cells exhibited a 49% reduction in TEER. This proves that DON alone was significantly reducing the strength of the cell monolayer, which would be consistent with previous studies [15,56,59]. After two hours of *E. coli* infection, there was a further reduction in TEER in both treatments. This negative effect of *E. coli* on TEER is well-established [27,50,60]. Two hours post-infection, cells pre-exposed to DON exhibited only 52% of the TEER of the *E. coli* treatment. Taken together, these results indicate that pre-exposing IPEC-J2 cells to DON increased the severity of barrier damage following *E. coli* infection.

*E. coli* cells attach to receptors on the surface of intestinal cells to colonize, release toxins, and exert inflammatory or barrier-disrupting effects [61]. Cells exposed to DON for 24 h before being infected with *E. coli* were more susceptible to *E. coli* adhesion relative to cells exposed to only *E. coli*. It is unknown by what mode of action DON increased *E. coli* adhesion, highlighting a potential area of future research. It is possible that DON could cause morphological changes to intestinal cells, increasing vulnerability to *E. coli* attachment [62,63]. In other investigations, DON has been shown to cause cell detachment from the monolayer, leaving more of the cell exposed to *E. coli* and making receptors more available [64]. There was no discernible detachment of cells exposed to DON in this study. However, the observed functional decreases in epithelial barrier integrity could make more surface area available on intestinal cells, leading to a DON-induced increase in *E. coli* adhesion. Future studies should investigate the effects of DON on glycoprotein and glycolipid receptors, which serve as the primary binding for *E. coli*, to better elucidate the underlying mechanisms [65,66].

## 4. Conclusions

Under the current experimental conditions, *E. coli* infection primarily accounted for the inflammatory and tight-junction protein changes seen in this study. However, pretreatment with DON prior to *E. coli* infection led to an increase in cell death, *E. coli* adhesion, and epithelial permeability. Taken together, this evidence suggests that pre-exposure to DON may exacerbate some response of IPEC-J2 cells to F18 ETEC. However, a major limitation of this study is that experiments were conducted in an in vitro system, and it is unknown what results may translate to animals. Therefore, additional experiments in pigs are needed to determine the effect of pre-exposure to DON on the response to *E. coli* infection in an animal system. Further, additional in vivo and in vitro studies could focus on how pre-exposure to DON prior to *E. coli* infection may affect additional parameters, such as oxidative stress.

## 5. Materials and Methods

### 5.1. IPEC-J2 Cell Culture

Experiments were conducted with IPEC-J2 cells that were originally isolated from the jejunum of a neonatal piglet and were cultured as previously described [67]. Cells were routinely cultured at 37 °C in 5% CO_2_. Cells were grown in basal medium composed of 93% Dulbecco’s Modified Eagle’s Medium F12 (DMEM F12 (Sigma-Aldrich, St. Louis, MO, USA), 5% fetal bovine serum (Thermo Fisher Scientific, Waltham, MA), 1% insulin–transferrin–selenium premix (Corning, Corning, NY, USA), 1% antibiotic–antimycotic mixture (Invitrogen, Carlsbad, CA, USA), and 5 ng/mL epidermal growth factor (EGF) (Sigma-Aldrich). One day prior to the start of an experiment, cells were seeded into plates at a density appropriate for attaining a confluent cell monolayer. Cells were 2–3 days post-confluence prior to use for experiments. After being thawed fresh from cryopreservation, cells were used for experiments within passages 10 and 20.

### 5.2. Experimental Model

There were four experimental treatments: Control, DON, *E. coli*, and DON + *E. coli*. On day one of the experiment, cells were seeded into plates at a density of 300,000 cells/mL. The following day, the DON and DON + *E. coli* treatment wells were exposed to 0.5 μM DON in basal growth media for 24 h. The Control and *E. coli* treatment received new media when other treatments were given DON-contaminated media. On day three of the experiment and upon the end of the 24 h DON pre-exposure, growth media were removed from all experimental wells, and cells were washed twice with 1× PBS. The media in the Control and DON treatments was replaced with basal media. Cells in the *E. coli* and DON + *E. coli* treatments were infected with *E. coli* in antibiotic/antimycotic-free media for 3 h for gene expression analysis. This 2 × 2 factorial arrangement was the experimental model for the measurement of gene expression, lactate dehydrogenase activity, ELISAs, and Western blot. Slight adaptations in the model were made for the measurement of translocation, adhesion, TEER, and FITC-dextran passage, which are noted in their respective methods’ descriptions.

### 5.3. Deoxynivalenol Conditions

Deoxynivalenol was obtained from Cayman Chemical, Ann Arbor, MI, USA. The DON dosage was optimized through preliminary experiments to find a concentration that did not cause detrimental cytotoxicity when combined with *E. coli*. The selected concentration of DON was 0.5 µM, and IPEC-J2 cells were exposed to DON for 24 h prior to the start of *E. coli* infection. The DON-contaminated media was removed from respective treatment wells and replaced with basal media when the *E. coli* treatments were administered.

### 5.4. F18 East 1 + E. coli Culture

The 3EC1 strain of F18 East 1 positive enterotoxigenic *E. coli* (F18-3EC1) used in this study was originally isolated from rectal swabs of pigs by Dr. Donald Bade in Ft. Collins, CO). On the day prior to each experiment, fresh *E. coli* was cultured from glycerol stock kept at −80 °C and diluted 1000-fold into brain–heart infusion (BHI) media (211059, Becton, Dickinson and Company, Franklin Lakes, NJ, USA) for an initial culture. On the day of infection, the initial culture was again diluted 1000-fold into BHI for 4 h to allow bacteria to reach mid-log phase, and this final culture was utilized for experimental infection. The bacteria culture was centrifuged to obtain a pellet, which was then washed twice with 1× phosphate-buffered saline (PBS) and resuspended in PBS prior to use for infection. The *E. coli* was then diluted into growth media without antibiotic/antimycotic and added to cells at a targeted multiplicity of infection of 5:1. The 5:1 multiplicity of infection was decided based on our preliminary data.

### 5.5. Epithelial Barrier Integrity Measurements

The measurement of *E. coli* translocation, adhesion, TEER, and 4-kDa fluorescein isothiocyanate-dextran (FITC-dextran, Sigma-Aldrich) passage involved two experimental treatments: *E. coli* and DON + *E. coli*. The DON exposure time remained consistent at 24 h. To identify whether pre-exposure to DON increased *E. coli* passage through the cell monolayer, a bacterial translocation assay was performed. Cells were seeded onto transwell inserts (3.0 µm pore size; Greiner Bio-One, Kremsmünster, Austria) at a density of 200,000 cells per well. The interior of the transwell insert contained 0.5 mL of treatment media, and the basolateral media had a volume of 1.5 mL of growth media. DON was placed in the apical and basolateral compartments during exposure. Following the DON challenge, treatments were apically exposed to *E. coli* for 1 h. At the end of the hour, 150 μL of basolateral media was collected. Basolateral media was serially diluted and plated onto MacConkey Sorbitol Agar (SMAC) in four replicates (M13-110, Alpha Biosciences, Baltimore, MD, USA). Colonies were grown overnight and then counted.

To understand how treatments impacted the strength of the epithelial barrier, TEER and FITC-dextran passage were measured. Cells were seeded onto transwell inserts (0.4 µm pore size, Greiner Bio-One, Kremsmünster, Austria) at a density of 200,000 cells per well and cultured as previously described. An EVOM2 Voltohmmeter (World Precision Instruments, Sarasota, FL, USA) was used to measure basal TEER value prior to *E. coli* infection (0 h) and 2 h post-infection (2 h). The TEER value used for analysis was corrected for basal TEER (membrane without cells). Furthermore, FITC-dextran was added in the apical media 3 h post-infection for 1 h (until 4 h post-infection). Basolateral media was then tested for the concentration of FITC-dextran through fluorometry with an excitation wavelength of 485 and an emission wavelength of 530 nm on a TECAN Spark 10M plate reader (TECAN, Zurich, Switzerland).

### 5.6. E. coli Adhesion Assay

The *E. coli* adhesion assay consisted of two treatments: *E. coli* and DON + *E. coli*. Cells were plated at a density of 300,000 cells/mL. Cells were exposed to DON for 24 h, followed by *E. coli* infection, which lasted for 30 min, after which cells were washed four times with 1× PBS and lysed with 0.5% Triton X buffer (Sigma-Aldrich, St. Louis, MO, USA). The remaining solution was plated on SMAC plates. Colonies were allowed to grow overnight prior to counting.

### 5.7. Real-Time qPCR

After 3 h of exposure to *E. coli*, total RNA was extracted from cells using TRIzol (Invitrogen, Waltham, MA, USA). The concentration and purity of RNA were measured using the Nanodrop ND-1000 spectrophotometer (Thermo Scientific, Waltham, MA, USA). Reverse transcription of 1 μg RNA was performed with MMLV reverse transcriptase to create cDNA (Promega, Madison, WI, USA). Quantitative real-time PCR was conducted with SYBR green RT-PCR mix (Bimake, Houston, TX, USA) using the CFX-96 real-time PCR detection system (Bio-Rad, Hercules, CA, USA). The mRNA expression of the following genes was determined to measure inflammatory or defense response (interleukin-6 (IL-6), interleukin-8 (IL-8), tumor necrosis factor alpha (TNFα) and beta-defensin 2 (β-defensin 2)); antioxidant response (glutathione peroxidase 1 (GPX1), nuclear factor erythroid 2-related factor 2 (NRF2) and superoxide dismutase 1 (SOD1)); and metabolic response (citrate synthase). The mRNA expression of these genes was normalized to the expression of 18S ribosomal RNA. The sequences of the primers involved are provided in Table 1.

### 5.8. Protein Expression Measured Through Western Blot

Upon termination of an experiment, cells were washed with 1× PBS and subsequently lysed in 1× radio-immunoprecipitation assay (RIPA) lysis buffer with 1% protease and phosphatase inhibitor cocktail (Thermofisher, Waltham, MA, USA). Prior to use, cell lysate was centrifuged at 12,000 rpm at 4 °C for 20 min to obtain supernatant free of cell debris. Lysate was then mixed with 4× SDS loading buffer (Boston Bio-Products, Inc., Milford, MA, USA) and denatured at 96 °C for 10 min. Total protein concentration was measured through a bicinchoninic acid assay kit (Sigma-Aldrich, St. Louis, MO, USA). Proteins were resolved on 10% SDS polyacrylamide gels and transferred onto nitrocellulose membranes (Bio-Rad, Hercules, CA, USA) with a semi-dry transfer system (Bio-Rad, Hercules, CA, USA). Membranes were subjected to 2 h of room temperature blocking with 5% BSA dissolved in Tris-buffered saline with 0.1% Tween. Following blocking, membranes were incubated with respective primary antibodies at a 1:1000 dilution overnight at 4 °C. The following primary antibodies were used: Beta-Actin Monoclonal Antibody (D6A8, Cell Signaling Technology, Danvers, MA, USA), Claudin 1 Polyclonal Antibody (51-9000, Invitrogen, Waltham, MA, USA), Claudin 4 Monoclonal Antibody (32-9400, Invitrogen, Waltham, MA, USA), and Anti-Occludin Polyclonal Antibody (ab31721, Abcam, Cambridge, UK). The next day, membranes were washed and incubated with secondary antibodies at a 1:25,000 dilution for at least 12 h at 4 °C. The secondary antibodies used for this study were anti-rabbit IgG, HRP-linked Antibody (#7074, Cell Signaling Technology, Danvers, MA, USA) and anti-mouse IgG, HRP-linked Antibody (#7076, Cell Signaling Technology, Danvers, MA, USA). Immobilon chemiluminescent HRP substrate (Millipore, Billerica, MA, USA) was utilized for blot development. Blots were then captured with a ChemiDoc imager (Bio-Rad, Hercules, CA, USA). ImageJ software was used to quantify blots (v1.53, NIH, Bethesda, MD, USA).

### 5.9. Protein Expression Measured with ELISA

The protein concentration of IL-8 was measured with the use of a porcine IL-8 ELISA kit (Catalog Number: ELP-IL8, RayBiotech, Peachtree Corners, GA, USA) on filtered cell culture media. Samples were measured in duplicate. The final absorbance was measured using a Tecan plate reader (Tecan, Zurich, Switzerland). A TNFα ELISA was also performed, but the levels were not detectable.

### 5.10. Lactate Dehydrogenase Assay

LDH activity was measured as an indicator of cellular cytotoxicity. The Takara LDH assay kit (Takara, Tokyo, Japan) was utilized to quantify LDH activity in cell culture media. A group of wells was treated with Triton X and used as a positive control for maximum LDH release. Relative cytotoxicity (%) was then calculated in reference to the positive control as instructed by the manufacturer.

### 5.11. Statistical Analysis

Results were determined through data from at least 3 independent experiments. The number of observations for all data are included in figure legends. The PROC-MIXED procedure of the SAS (version 9.4) software was used. Tukey’s test for multiple comparisons was performed to identify differences between means of treatments. The Shapiro–Wilk test was used to assess normality. When normality could not be obtained, a Kruskal–Wallis test was performed. *p*-values < 0.05 were considered significant, while those between 0.05 and 0.1 were considered a tendency. The experimental unit was the well of the plate. The fixed effects were DON and *E. coli* inclusion for ELISA, qPCR, Western blot and LDH assay. The fixed effect for translocation, adhesion, FITC-dextran and TEER measurements was treatment. Replication was used as a random effect. If there was not a significant interaction, the interaction term was removed, and main effects were presented. Graphics were created through Excel.

## Figures and Tables

**Table 1:** Primers used for qPCR.

Gene	Forward Primer (5′–3′)	Reverse Primer (5′–3′)	Accession Number
18S	ATCCCTGAGAAGTTCCAGCA	CCTCCTGGTGAGGTCGATGT	XM_059255789.1
IL-8	AGAGGTCTGCCTGGACCCCA	GGGAGCCACGGATGGGT	NM_213867.1
IL-6	TTCACCTCTCCGGACAAAAC	TCTGCCAGTACCTCCTTGCT	NM_214399.1
TNFα	CTACTGCACTTCGAGGTTATC	GGGCTTATCTGAGGTTTGAG	NM_214022.1
β-Defensin 2	GACTGTCTGCCTCCTCTC	GGTCCCTTCAATCTGTTG	AY506573.1
GPX1	TACAGCCGTCGCTTTCTGAC	CACTCTAGGCACTGCTAGGC	NM_214201.1
NRF2	TGTCTTTGGATTTAGCGTTTCGG	TCCATGTCCCTTGACAGCAA	XM_021075133.1
SOD1	GTTGGAGACCTGGGCAATGT	TCAGACCATGGCATGAGGGA	NM_001190422.1
Citrate synthase	TGCCATGGCCTTACTCACTG	GGCAGCAAGAACAAGACAGG	NM_214276.1

## Data Availability

The original contributions presented in this study are included in the article. Further inquiries can be directed to the corresponding author.

## Abbreviations

The following abbreviations are used in this manuscript:

DON	Deoxynivalenol
ELISA	Enzyme-linked immune absorbent assay
ETEC	Enterotoxigenic *E. coli*
TEER	Transepithelial electrical resistance
PCR	Polymerase chain reaction
IPEC-J2	Intestinal porcine epithelial cells (jejunum);
EGF	Epidermal growth factor
DMEM	F12, Dulbecco’s Modified Eagle’s Medium F12
BHI	Brain–heart infusion
PBS	Phosphate-buffered saline
FITC-dextran	FITC-dextran, fluorescein isothiocyanate-dextran
LDH	Lactate dehydrogenase
BSA	Bovine serum albumin
GPX1	Glutathione peroxidase 1
NRF2	Nuclear factor erythroid 2-related factor 2
SMAC	MacConkey Sorbitol Agar
IL	Interleukin
TNF	Tumor necrosis factor
RIPA	Radioimmunoprecipitation assay buffer
SOD1	Superoxide dismutase 1
